# Ventral-subgenual anterior cingulate cortex and self-transcendence

**DOI:** 10.3389/fpsyg.2013.01000

**Published:** 2013-12-27

**Authors:** Yi-Yuan Tang, Rongxiang Tang

**Affiliations:** ^1^Department of Psychology, Texas Tech UniversityLubbock, TX, USA; ^2^Department of Psychology, University of OregonEugene, OR, USA; ^3^Department of Psychology, The University of Texas at AustinAustin, TX, USA

**Keywords:** self-transcendence, altered state of consciousness, anterior cingulate cortex, meditation, integrative body–mind training

Self-transcendence (ST) is one of specific human experiences often related to harmony with nature or feeling oneness with others or the self as an integral part of the whole universe. The Temperament and Character Inventory (TCI) is a widely used personality measure, and ST is one of personality dimensions (Cloninger, 1994; Cloninger et al., 1994). Previous studies showed that ST has significant positive correlation with the sgACC encompassing a ventromedial portion of the prefrontal cortex (vmPFC) using TCI and PET scan (Hakamata et al., 2013). Meanwhile, sgACC/vmPFC activity has been shown to be significantly decreased in patients with anxiety, major depression and mood disorders (Drevets et al., 2008; Shin and Liberzon, 2010; Kühn and Gallinat, 2013). Altogether, these findings suggest that sgACC/vmPFC play an important role in emotion regulation and ST (Hakamata et al., 2013).

ACC as a part of the brain's limbic system, appears active in many neuroimaging studies (Bush et al., 2000; Posner et al., 2007). In general ACC is involved in cognitive (dorsal division) and emotional (ventral/rostral part) processing (Bush et al., 2000). The sensitivity of the ACC to both reward and pain, and evidence for ACC coupling to cognitive and emotional areas during resting state and task performance, support the role of ACC in self-regulation or self-control including emotional, cognitive and autonomic control. Particularly, v/sgACC and adjacent mPFC area involves in emotional control and autonomic regulation (Luu and Posner, 2003; Posner et al., 2007), consistent with many meditation findings (Hölzel et al., 2011; Tang et al., 2012b; Tang and Posner, 2013).

Meditation often exemplifies positive emotion, pleasant feeling and ST experience in practitioners (Cahn and Polich, 2006; Tang et al., 2007). Studies showed ST is positively related to meditation practice (Levenson et al., 2005). One meditation-category—automatic self-transcending includes techniques designed to transcend their own activity and improve ST (Travis and Shear, 2010). Substantial evidences indicate that ACC plays a key role in meditation training (Hölzel et al., 2011). For example, compared to non-meditators, long-term Vipassana meditators showed stronger activations in the rostral ACC and adjacent medial PFC bilaterally for the meditation condition (contrasted to arithmetic task). Greater ACC and mPFC activations in meditators may reflect processing of distracting events and emotional processing (Hölzel et al., 2007, 2011). Compared with a memory training control, compassion training elicited activity in a neural network including pregenual ACC, medial orbitofrontal cortex and striatum—brain regions previously associated with positive affect and affiliation (Klimecki et al., 2013a,b). In the same vein, 5 days of one form of meditation—integrative body–mind training (IBMT) improves vACC activity compared to same amount of relaxation training (Tang et al., 2009). Meanwhile, 5 days of IBMT also reduces stress, improves positive emotion and self-report of feeling oneness with nature (Tang et al., 2007). Further, 10 days of IBMT increases white matter connectivity surrounding ACC and this brain structural change correlates with emotional regulation (Tang et al., 2012a,b). These results indicate that meditation accompanies positive emotion, ST experience, and ACC functional and structural changes.

ST related meditation not only induces brain and behavioral changes, it often involves brain (mind) and body cooperation indexed by central (CNS) and autonomic (ANS) nervous system interaction (Cahn and Polich, 2006; Hölzel et al., 2011). Studies have begun to explore interaction and dynamics between CNS and ANS (Critchley et al., 2003; Tang, 2009; Tang and Posner, 2009; Tang et al., 2009; Hölzel et al., 2011; Critchley and Harrison, 2013). For instance, using heart rate variability (HRV), and high- and low-frequency power in the cardiac rhythm, ACC activity related to sympathetic modulation of heart rate was observed (Critchley et al., 2003). We measured the physiological and brain changes at rest before, during, and after 5 days of IBMT and relaxation training. During and after training, the IBMT group showed significantly better physiological reactions in heart rate, respiratory amplitude and rate, and skin conductance response (SCR) than the relaxation control. Differences in HRV and EEG power suggested greater involvement of the ANS in the IBMT group during and after training. Imaging data demonstrated stronger v/sgACC activity in the IBMT group. Frontal midline ACC theta was also correlated with high-frequency HRV, suggesting control by the ACC over parasympathetic activity (Tang et al., 2009). These results indicate that brief IBMT induces better regulation of the ANS by a midline v/sg ACC brain system. This changed state probably reflects training in the coordination of body and mind given in the IBMT but not in the relaxation group. These results indicate body-brain works together to maintain certain consciousness states such as ST that may be related to different performance (Tang et al., 2007; Xue et al., 2011; Tang et al., 2012a,b). Our findings suggest meditation training could induce altered states of consciousness which may allow us to explore the neuroscience of consciousness based on how alterations in normal consciousness result in functional or/and structural brain changes and plasticity. These alterations in consciousness can affect long-term cognitive, affective and social activities, and may help understand the disease states or disorders of consciousness such as coma, vegetative state, etc. (Tang et al., 2013).

In summary, growing empirical evidences indicate meditation has potential to develop ST—a positive relationship between self and other that transcends self-focused needs and increases prosocial characteristics (Hölzel et al., 2011; Tang et al., 2012a,b; Vago and Silbersweig, 2012). Future studies could examine the relationship between ST and short-term or long-term meditation, and how meditation shapes the perspectives on the self, self-others, self-nature and its underlying mechanisms using multimodal neuroimaging, physiological, psychosocial and genetic methods.
